# A Therapeutic Matrix: Virtual Reality as a Clinical Tool for Spinal Cord Injury-Induced Neuropathic Pain

**DOI:** 10.3390/brainsci11091201

**Published:** 2021-09-12

**Authors:** Erik Leemhuis, Valentina Giuffrida, Anna Maria Giannini, Mariella Pazzaglia

**Affiliations:** 1 Department of Psychology, Sapienza University of Rome, Via dei Marsi 78, 00185 Rome, Italy; erik.leemhuis@uniroma1.it (E.L.); valentina.giuffrida.gv@gmail.com (V.G.); annamaria.giannini@uniroma1.it (A.M.G.); 2 Body and Action Lab, IRCCS Fondazione Santa Lucia, Via Ardeatina 306, 00179 Rome, Italy

**Keywords:** virtual reality, spinal cord injury, neuropathic pain, rehabilitation, disembodied, neuroplasticity, deafferentation, body representation

## Abstract

Neuropathic pain (NP) is a chronic, debilitating, and resistant form of pain. The onset rate of NP following spinal cord injuries (SCI) is high and may reduce the quality of life more than the sensorimotor loss itself. The long-term ineffectiveness of current treatments in managing symptoms and counteracting maladaptive plasticity highlights the need to find alternative therapeutic approaches. Virtual reality (VR) is possibly the best way to administer the specific illusory or reality-like experience and promote behavioral responses that may be effective in mitigating the effects of long-established NP. This approach aims to promote a more systematic adoption of VR-related techniques in pain research and management procedures, highlighting the encouraging preliminary results in SCI. We suggest that the multisensory modulation of the sense of agency and ownership by residual body signals may produce positive responses in cases of brain-body disconnection. First, we focus on the transversal role embodiment and how multisensory and environmental or artificial stimuli modulate illusory sensations of bodily presence and ownership. Then, we present a brief overview of the use of VR in healthcare and pain management. Finally, we discus research experiences which used VR in patients with SCI to treating NP, including the most recent combinations of VR with further stimulation techniques.

## 1. Introduction

Pain management remains one of the biggest challenges in healthcare, mostly due to the uncertain results of drug therapies and their many undesirable side effects [1]. Pain is defined as “an unpleasant sensory and emotional experience associated with or described in terms of actual or potential tissue damage” [2]. Those who live with pain may give more emotional descriptions, defining it as an experience that detaches them from life and makes everything difficult or impossible. Individuals with spinal cord injuries (SCI) are particularly sensitive to this issue. Approximately one-third of people with SCI experience persistent chronic pain, ranking it as severe or excruciating within 1 year after the SCI [3,4]; even after recovery and without a visible cause, the pain can itself become a disease.

Neuropathic pain (NP) is among the most prevalent forms of chronic pain in patients with SCI and appears to be particularly difficult to resolve [5,6]. NP may occur above, below, or at the level of the lesion. Opioid medications are too often the first or only pharmacological option, but over both the short and the long term the side effects may be as detrimental as the pain [1].

At-level NP is perceived in the same area where the lesion occurred. In this case, pain is identified in a segmental pattern anywhere within the dermatome at the neurological level of injury or within the three dermatomes below this level. If the former condition is the case, the pain can be classified as at-level NP even if it is found in one dermatome above the neurological level of the lesion [7]. It usually emerges soon after the SCI and includes central and peripheral components [5]. Below-level pain is generally of central origin and appears when chronicity has set [4,5,8], and refers to NP perceived in more than three dermatomes below the neurological level of the lesion [9]. Above-level NP is pain perceived above, at, or below the level of lesion. Finally, pain not related pathologically to the SCI is known as “other neuropathic pain” [7].

What triggers maladaptive results such as NP in the reorganization process remains mostly unclear, making treatment difficult. Recently, the role of neuroimmune dysregulation has been considered central to the occurrence of NP caused by early inflammatory response to SCI. This response is necessary for tissue recovery at the injury site during the acute post-SCI stages, but may result in scars that prevent proper functional regeneration and may negatively contribute to central maladaptive neuroplasticity and chronic pain [5]. Drugs to prevent the insurgence of chronic pain are not yet available. In clinical practice, substances such as methylprednisolone are often used but are known to produce numerous side effects, such as gastrointestinal bleeding or greater susceptibility to infections, yielding uncertain results [10,11].

In light of the above, and because in many patients pain persists despite the use of drugs, non-pharmacological approaches to chronic pain relief are increasingly sought. Such therapies are based on the principle that the pathophysiological consequences of such cortical plasticity may underlie the development of phantom sensations and pain [12,13,14,15] by targeting somatosensory neural reorganization after injury. Therefore, dealing with neuropathic pain remains a major challenge. One of the most recent non-pharmacological and non-invasive intervention tools for neuropathic pain is the use of virtual reality (VR). VR is a technology that is being used increasingly in numerous applications in the clinical field and shows great potential [16,17].

This study aims to present an overview of the role that VR could have in the management of chronic pain and PN. It focuses on the transversality of the sensory, motor, environmental, and technological factors involved. The key factors in the construction of body awareness and embodiment processes are presented in the next section to show how VR exploits these mechanisms to generate and modulate illusory sensations of bodily presence and ownership. The third section offers insights into how VR is entering clinical practice in numerous ways and then examines pain and its management through VR. The fourth section describes the specifics of patients with SCI and the use of virtual experiences in treating NP, and includes the most recent combinations of VR with further stimulation techniques.

## 2. “It’s My Body!” The Use of VR as an Embodiment Tool

Embodiment consists of two components: the sense of ownership (understood as the feeling that a certain body part or artificial agent belongs to us as part of our body) and the sense of acting (understood as the feeling that our body is responsible for its movement) [18,19]. Producing specific changes in the subjective experience of embodiment may produce alterations in bodily sensations, interactions with the environment, and, in certain conditions, reduce pain intensity [20,21,22,23]. This process results from integrating all sensory afferents with memory and ongoing cognitive and affective activities [24]. By exploiting all of the factors that underpin embodiment, VR effectively produces illusory alterations in body ownership, promoting the embodiment of a virtual body (i.e., an avatar) or of single body parts [25,26,27].

VR can be defined as a technology that allows the creation of an artificial or virtual environment that simulates reality [28]. The use of proper devices allows different degrees of immersion and interaction of the user in a virtual scene. This perceived control enhances the subject’s sense of “presence” in the virtual environment. Depending on the level of immersion, three main categories of VR can be defined. Personal computers, tablets, or smartphones are used for non-immersive VR, meaning that the subject maintains a degree of contact with reality. Semi-immersive VR uses large screens coupled with relatively high-performance graphics to induce a deeper feeling of immersion in the virtual environment. Owing to a head-mounted display (HMD), immersive VR allows a strong sense of agency for users, excluding most real-world visual and auditory stimuli [28]. Immersive VR offers an innovative and valuable resource for pain management, as it not only provides more intense interaction and a greater sense of presence (i.e., the perception of being in the “virtual room”) but also facilitates a greater illusion of owning the virtual body, highlighting the importance of the relationship between the illusion of having a body and the decline of perceived pain [20,21,29,30].

The brain uses representations of the body (i.e., body image and body schema) for probably all “top-down and bottom-up” processes, all of which rely on sensory and motor signals that verify ownership of the body [31,32]. To be aware of our body and our actions, we need correspondence among the visual, tactile, and proprioceptive signals from the various parts of our body, which contribute to an intermodal sensory body image [18]. Subsequently, the correspondence between intentions and their effects on the body through the generation of actions contributes to a sense of the self as having agency [18,33]. The best way to define body awareness may be as the result of the dynamic integration of multisensory bodily signals [34] and salient information about the surrounding space (i.e., peripersonal space) [35,36,37].

VR facilitates modulating our perceived sense of ownership of the whole body [25,26] or of single body parts [38] by altering the congruity of environmental and bodily multisensory information [39,40,41]. Research exploring the different contributions to the intensity of induced embodiment started decades ago. Hoffman et al. sustained the importance of multiple sensory inputs such as olfactory signals and tactile feedback to increase the sense of presence [42]. Subsequently, a study reported that the control of movement was more essential than the enrichment of the virtual scene, guaranteeing a greater sense of presence for the subject and shifting the emphasis to the level of interaction/interactivity [43]. In this sense, one of the strengths of immersive or semi-immersive VR lies precisely in the creation of a condition in which the subject is no longer a mere external spectator but an actor in a condition of complete sensory immersion [44].

## 3. VR for Real Relief

In light of the above and despite the many open questions on current understanding of the processes involved in the use of VR, it is easy to foresee its potential and ease of use. The possibility of reproducing any type of environment and situation in the healthcare and laboratory contexts, together with the ability to manipulate the user’s perceived body characteristics, can attract the interest of numerous aspects of clinical practice. The use of experimental VR procedures in a wide range of cognitive and physical rehabilitation programs yields very interesting results. VR has proven to be an effective tool in assessing cognitive functions owing to the validation of tasks carried out in VR for the assessment of memory and attention [45,46,47]. Assessment interviews administered by an avatar in a virtual environment using an HMD on patients with dementia may facilitate cognitive assessment [48]. In another study on patients with dementia, VR enhanced pleasure and increased vigilance during and after exposure to pleasant environments via an HMD [49]. In patients suffering from stroke [50,51], cerebral palsy [52], and SCI [53,54], VR produced improvements in gait and balance. In subjects with traumatic brain injury, VR may improve physical and cognitive functions [55].

One of the greatest promises of VR consists of the possibility to systematically introduce it into clinical practice as an effective non-pharmacological tool for pain management [1,56,57,58]. The sense of ownership “of the avatar as one’s own body” produced by multisensory stimulation [22,23] and the sense of perceived control are probably the biggest factors that explain increased pain tolerance [23,59]. In particular, the perception of control positively influences pain tolerance and promotes adaptive behaviors, beliefs, and coping strategies [60].

Pain-imaging studies have identified the insular cortex, secondary somatosensory cortex, and anterior cingulate cortex as part of the pain matrix, which informs the body about the intensity and location of the noxious stimulus present [59,61]. Based on this, a functional imaging study by Salomons et al. (2004) showed that the perception of controlling the pain stimulus causes a change in neuronal activation in regions of the pain matrix, which present an attenuated activation [62]. The results confirmed that perceived control reduces both the subjective experience of pain [63] and the neuronal responses. One reason for this may be the subject’s reappraisal of less threatening pain, occurring because the activation of dorsal prefrontal regions is involved in the voluntary reappraisal and high-level appraisal processes, effectively reducing the intensity of the pain [64].

The embodiment effect of VR and its efficacy on pain probably share certain principles with other procedures, such as the mirror box technique. The use of mirror visual feedback (MVF) was shown to reduce pain intensity and perceived unpleasantness [22], probably opposing the effects of maladaptive neuroplastic changes and central regulatory effects [65]. This finding supports the idea that viewing one’s own healthy “functioning” body induces changes in the cortical representations where pain signals are processed, resulting in a modulation of pain perception of the affected body part [22,23].

These early insights motivated subsequent studies that increased the use of VR (which can effectively extend the MVF technique), with all the advantages that VR possesses [17], such as increased user involvement and motivation [66,67,68] and compliance with long-lasting rehabilitation programs [69,70]. The analgesic effect from the use of VR in the treatment of pain can often be derived from the effect of “distraction,” moving the user’s attention away from the physical body and pain [71,72]. However, virtually restoring visual feedback in response to efferent cortical motor signals or visuotactile stimulation likely explains a large part of the positive effect of VR treatments. This, as seen with the MVF technique, could affect the degree of reorganization of somatosensory pathways [73], and the pain reduction achieved with MVF may be a clue to the partial reversal of maladaptive reorganization [65].

## 4. VR as a Pain Management Technique in SCI

Pain reduction of the type seen in multisensory VR exposure induces or facilitates neuronal plasticity processes [74]. NP may be attributed to neuroplastic reorganization, and several protocols in VR are being used to reverse maladaptive plasticity and effects on the somatosensory and motor cortex after SCI [75,76,77,78,79,80,81]. Activity in S1 and M1 (which are functionally closely linked) probably holds the most precise representation of the felt body [82,83,84,85,86], concurring with specific adaptive cortical change [76,77,78,79,80,81]. Thus, a frequently used method to reduce NP after SCI is the virtual walking technique [58,68,69,87,88,89]. In this technique, the subject imagines the movement. Motor imagery is the mental execution of a movement, and seems to share similar underlying mechanisms to real movement [90,91,92]. The same occurs in the behavioral domain: a movement phenomenon has mental isochrony, meaning the time required to perform a movement is the same even when the movement is imagined [93].

An analgesic response to VR combined with virtual walking, independent of the lesion level, was observed up to three months after the rehabilitation program, suggesting neuroplastic changes. Despite the non-immersive VR experience, the patients’ affected body aligned to the lower body with normal walking ability, and patients reported a good degree of body illusion [87]. This suggests that VR, as in mirror therapy [65], acts on pain mainly via visual capture, partially restoring the lost coherence between body motor output and sensory feedback. The pre–post comparison indicates that imagining oneself walking has short-term positive and long-term analgesic effects on NP.

These findings make it difficult to untangle the structural effect only from changes that cause pain; however, they clearly demonstrate that the analgesic effect of multiple or longer sessions is facilitated by an intact virtual sensorimotor loop. Regarding plasticity mechanisms, up-regulation of motor cortex excitability by non-invasive stimulation techniques [58,88,94,95,96,97] combined with VR has clearly shown analgesic effects.

Specifically, two studies that used the combination of transcranial direct current stimulation (tDCS) and virtual walking [98,99] showed the positive effects of motor stimulation both immediately after the end of the treatment and up to twelve weeks following treatment. Compared with tDCS alone, combined stimulation and visual illusion of the body yielded better synergistic effects of the intervention and a longer duration of analgesic benefits. Patients reported a mean 50% decrease in the numerical pain rating scale score for NP after VR-tDCS. The efficacy of neuromodulatory therapy, combining VR and anodic tDCS over the motor cortex, has also been studied on different subtypes of pain that can reflect different pathophysiological mechanisms [100]. Combined stimulation is more effective in paroxysmal pain, mechanical allodynia, and dysesthesia, with positive effects for up to four weeks after treatment [99].

Recently, two studies investigated the effect of tDCS combined with visual illusion according to sensory phenotype profiles of NP [101,102]. Treatment resulted in a significant reduction in pain and pain-related sleep disturbances associated with NP. For example, pain resulting from burns, squeezing, electric shocks, stabbing, pins, needles, and tingling significantly improved. However, no significant phenotypes were identified [97].

Theoretically, the stimulation of cortical activity using neuromodulatory techniques could promote plasticity and enhance pain reduction [99,103,104,105]. Primary motor cortex stimulation using tDCS modulates cortical excitability [105], which may result in a reduction of NP with long-lasting effects. Therefore, the combination of tDCS and VR is very promising and warrants extensive investigation.

Motor imagery is a promising technique for motor rehabilitation [93,106,107] and can enhance the effects of VR activities, increasing the sense of embodiment and favoring adaptive plasticity. Its effects on motor function have been attributed to the strengthening of motor programs [93,108,109], while its effects on pain remain unclear. It has been hypothesized that the production of motor imagery may influence the interaction among mental representations of the body, nociception, sensorimotor integration, and pain [110,111]. Compared with wheelchair wheeling, virtual walking showed a mild but significant reduction in NP after SCI [69].

However, in SCI cases the movement imagery obtained contradictory results, which prevents us from drawing certain conclusions [112].

The critical importance of owning and sensing a virtual body is best explained using multisensory body illusions (i.e., full-body illusion and virtual leg illusion) combining visual and tactile inputs [20,25,37,113,114,115]. The effects of VR on chronic pain showed that in a condition of visual-tactile congruence, subjects exhibited a higher degree of embodiment of the virtual body and lower pain perception. Pozeg et al. specifically treated NP in SCI subjects using both synchronous and asynchronous tactile stimulation conditions with full body and virtual leg illusions. The results showed that in the full-body condition, there was a decrease in pain in both synchronous and asynchronous conditions. Interestingly, the virtual leg illusion generated a weaker sense of ownership in the SCI group than in the healthy control group, suggesting that sensorimotor disconnection plays an important role in sense of presence and the embodiment process for the purposes of pain reduction [113]. To preserve body image, embodiment may act as a means of preventing maladaptive plasticity and thereby preventing and managing refractory NP.

The use of VR in combination with coherent sensory feedback on the body has great potential for identifying and enhancing residual sensory and motor skills in patients with SCI. VR can provide an interactive simulated environment to allowing patients with somatosensory deficits to experience greater levels of bodily sensation to compensate for the lack of signals from missing limbs [71].

Another aspect to consider is the use of immersive VR from the first- [69,115] or third-person perspectives [87]. Although beneficial effects were found in both conditions, similar pain reduction in studies using a third-person perspective required more VR sessions. This could be explained as the increased feeling of embodiment allowing the subject to reduce the gap between sensory input and motor output, making the experience fully immersive. The high level of presence associated with first-person simulations is strongly associated with decreased pain [30,116,117]. This is probably due to the high distractive capacity of VR, which in the case of first-person perspective enhances the “disembodying” effects of the painful body toward the presence in the virtual environment.

Another combined treatment that has proven effective in reducing pain caused by burns [118] and physical trauma [119,120,121] is VR hypnosis (VRH). A case report on a patient with SCI found that 33 sessions of immersive VRH over six months allowed the subject to isolate themselves from the outside world and had positive effects on perceived pain intensity and unpleasantness. Interestingly, the duration of pain relief increased during the program from 3.9 h to 12.2 h after the last VRH session [122]. Furthermore, absorption (as measured by the Tellegen Absorption Scale [123]) showed a correlation between successful hypnotizability and imaginative immersion. Such an effect could be due to the shifting of attentional pathways away from pain; importantly, the effect can be potentiated over time. Attention is already known as a modulating factor in the perception of pain [124,125,126,127,128], and the synergy between VR and hypnosis may exploit this effect and shift attention away from the body, producing an “unlearning” effect towards pain.

Attention modulates pain perception [72,124,125,126,127,128,129], and VR strongly modulates attention in turn by prompting a high level of engagement. Thus, an experience combining VR with hypnosis is believed to optimize the effect of illusion and the sense of presence, increasing the level of distraction from the painful body enough to provide relief [122,130].

Furthermore, the results suggest that the greater the number of sessions, the greater the reduction in pain, which supports the need to plan long-lasting therapeutic interventions. Further comparisons and testing should be conducted to verify first- or third-person perspectives.

## 5. Conclusions

Despite considerable variability in procedures and results, current evidence is encouraging in terms of the effectiveness of simple or combined VR procedures on chronic pain. This is a promising starting point for further verification of the immediate analgesic effect and probable long-term beneficial effects. VR therapy is a viable, non-pharmacological, and non-invasive alternative for post-SCI NP, has minimal side effects, and has been prescribed over time. Body illusions and motor imagery combined with neuromodulatory therapy constitute representative experimental paradigms to successfully induce analgesia. The sense of embodiment has demonstrated efficacy in modulating both acute and chronic pain perception irrespective of the location of the pain. VR treatment attempts to reinsert the body of patients with SCI into the flow of experience, leading to better functional outcomes. Most studies so far have focused on paradigms which empower the embodiment process in individuals with intact bodies, rather than considering bodies with sensorimotor disconnection and residual signals. Therefore, after SCI the illusory ownership evoked during the RHI does not occur when stimulation is applied to numb body parts. Body parts with normal or residual tactile sensations appear to be an alternative for remapping input in affected body parts. It may be advantageous to adapt such techniques to include somatotopically focal stimulation of non-affected body parts to boost embodiment [131,132,133]. Other signals, in addition to tactile signals, could serve as mediators. Further studies are warranted to overcome the limitations of the current literature. Problems such as non-uniform protocols, VR experiences that cannot be compared, and small experimental groups are particularly common in this field. Moreover, specific to clinical groups such as SCI, aspects such as time since injury, type and level of lesion, and residual sensorimotor abilities are often not adequately assessed. Another complicating factor—the experience of pain—is a multidimensional process with different mechanisms resulting in a wide variety of pain symptoms. Although the actual mechanisms underlying these effects remain to be elucidated, consideration of an embodied virtual body in the interplay between sensory and cognitive–affective mechanisms opens the possibility of modifications that target body perception disturbances. Longitudinal studies will be essential to comprehend NP, and with easily available VR, such results will probably be readily achievable.
